# 571. Opioid-based Pain Management in Burn Surgery Patients with Substance Abuse

**DOI:** 10.1093/jbcr/irag033.311

**Published:** 2026-04-06

**Authors:** Armen Brotgandel, Carson Bair, Zayd Chishti, Ryan Hoffman, Sai Ram Velamuri

**Affiliations:** University of South Florida Health Morsani College of Medicine, Tampa, FL; Tampa General Hospital Regional Burn Center, Allentown, PA; University of South Florida Health Morsani College of Medicine, Tampa, FL; University of South Florida Health Morsani College of Medicine, Department of Plastic Surgery, Tampa, FL; University of South Florida Health Morsani College of Medicine, Tampa, FL

## Abstract

**Introduction:**

Pain management is challenging for patients with a history of substance abuse due to provider bias, risk of diversion, and altered neural/biochemical pain response. Surgically treated burn patients require aggressive pain management, but many patients present with a history of substance abuse. This retrospective study seeks to quantify pain management requirements in this population that disproportionately suffer from burns.

**Methods:**

All patients 18 years and older who underwent excision of burn wounds between Jan 2023 and Jun 2024 at a verified burn center were included (n = 111). History of substance abuse was self-reported or determined by urine toxicology. Marijuana use alone was not included as substance abuse. Total surgeries, length of stay, TBSA burnt, and morphine equivalents given throughout the entire admission were recorded. Patients were analyzed based on the number of operations required during admission to account for greater pain control needs. Outcomes were compared via Mann–Whitney U Tests, with *p*<.05 indicating statistical significance.

**Results:**

Of 111 patients, 28 had a history of substance abuse and 83 had no reported substance abuse. The most common substances abused included cocaine (n = 12), methamphetamines (n = 10), and IV opioids (n = 7). Patients with substance abuse had statistically significantly increased % body surface area burnt and non-significantly longer stays in hospital. Overall, those with a history of substance abuse required significantly more morphine equivalents. The same trend was seen for patients undergoing 1 surgery.

**Conclusions:**

Burn patients show a high rate of substance abuse (25% in the present study), and this population suffered from more severe burns requiring more intensive care. Patients with a history of substance abuse required more morphine equivalents for effective pain management. This difference can potentially be attributed to more severe burns in this patient population or increased tolerance. Future research should investigate how other factors including burn mechanism or location impact pain management.

**Applicability of Research to Practice:**

This data demonstrates that substance abuse can be a guiding factor in pain management in burn patients. Therefore, collecting an accurate social history is critical for effective pain management to optimize patient outcomes.

**Funding for the study:**

N/A.

